# Gas Gangrene in Orthopaedic Patients

**DOI:** 10.1155/2013/942076

**Published:** 2013-10-28

**Authors:** Zhimin Ying, Min Zhang, Shigui Yan, Zhong Zhu

**Affiliations:** ^1^Department of Orthopaedic Surgery, The Second Affiliated Hospital, School of Medicine, Zhejiang University, No. 88 Jiefang Road, Hangzhou, Zhejiang 317000, China; ^2^Taizhou Hospital of Zhejiang Province, The Forth Affiliated Hospital of Wenzhou Medical University, No. 150 Ximen Road, Linhai, Zhejiang 317000, China; ^3^Department of Orthopaedic Surgery, Taizhou Hospital of Zhejiang Province, The Forth Affiliated Hospital of Wenzhou Medical University, No. 150 Ximen Road, Linhai, Zhejiang 317000, China

## Abstract

Clostridial myonecrosis is most often seen in settings of trauma, surgery, malignancy, and other underlying immunocompromised conditions. Since 1953 cases of gas gangrene have been reported in orthopaedic patients including open fractures, closed fractures, and orthopaedic surgeries. We present a case of 55-year-old obese woman who developed rapidly progressive gas gangrene in her right leg accompanied by tibial plateau fracture without skin lacerations. She was diagnosed with clostridial myonecrosis and above-the-knee amputation was carried out. This patient made full recovery within three weeks of the initial episode. We identified a total of 50 cases of gas gangrene in orthopaedic patients. Several factors, if available, were analyzed for each case: age, cause of injury, fracture location, pathogen, and outcome. Based on our case report and the literature review, emergency clinicians should be aware of this severe and potentially fatal infectious disease and should not delay treatment or prompt orthopedic surgery consultation.

## 1. Introduction

Emergency physicians and surgeons are confronted with patients of gas gangrene so uncommon in civilian practice that many are unfamiliar with its signs and symptoms and do not recognize its development quickly and accurately. The difficulties in diagnosis not only lie in unfamiliarity with the signs and symptoms of gas gangrene but also in the lack of differentiation between contamination and infection and to the confusion between gas gangrene and various clostridial infections and other bacterial and nonbacterial lesions simulating gas gangrene [1, 2]. Gas gangrene occurs in a variety of clinical settings that can be subdivided into three major types: posttraumatic origins, postoperative origins, and spontaneous occurrences. Clostridial myonecrosis, also known as true gas gangrene, is the most devastating kind of clostridial infection which requires aggressive, early surgical management. Its onset is insidious and subsequent progressive rapidly. Spontaneous types occurred in patients with compromised medical conditions including uncontrolled diabetes mellitus and various forms of malignancy, the more commonly reported being leukemia and breast cancer [3]. *Clostridium septicum* is the major cause of nontraumatic spontaneous gas gangrene in patients with immunosuppressant diseases [4]. 

## 2. Case Report

A 55-year-old female obese farmer (BMI 35) presented to the Emergency Department with a two-day history of the right leg progressing sensory deprivation and swelling. Eight days ago she had a car accident which caused her right tibial plateau fracture. Two days after her hospitalization, elective surgery for the fracture was performed at a local hospital. Afterwards, the patient felt increasing pain out of proportion to physical findings accompanied by progressive swelling, numbness, and weakness of the limb. She was unable to move her right lower extremity and had no sensation below the knee joint level. These signs were not taken seriously. Then she was transferred to our university hospital with the presumptive diagnosis of gas gangrene. She denied chills, anorexia, or other symptoms. She also reported no previous episodes or other recent illnesses. Previous medical history included mild type 2 diabetes mellitus which lasted over 20 years poorly controlled with metformin and hypertension treated with hydrochlorothiazide. She was not taking any other medications.

**Figure 1:**
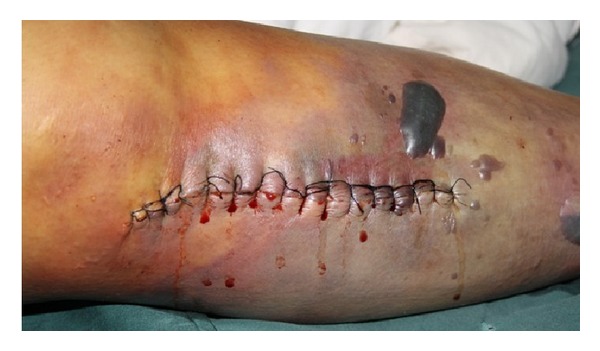
The limb was severe swollen and skin was brownish with bullae exuding from the incision.

On physical examination this patient was febrile, alert, and comfortable; vital signs were temperature of 38.2 degrees centigrade, pulse of 110 beats/min, respiratory rate of 18 breaths/min, and blood pressure of 105/57 mmHg. Head, neck, cardiac, lung, and abdominal examinations were unremarkable. The physical examination revealed severe swollen and brownish skin of the limb with bullae exuding from the incision (Figure 1). Subcutaneous crepitus extended along the length of the limb and the skin discoloration spread from knee to ankle. Roentgenograms revealed gas in the interfacial planes of the leg. Extensive gas formation throughout all the muscle compartments of the right leg reaching to the level of knee joint was present. Laboratory evaluation showed that white blood cell count was 22.2 × 10^9^/L, hemoglobin was 83 g/L, platelet count was 183 × 10^9^/L, a serum glucose of 324 mg/dL, C-reactive protein was 48.0 mg/dL, and serum creatinine was 155 *μ*mol/L. Examination of a needle aspirated from the incision showed gram-positive bacilli.

**Figure 2:**
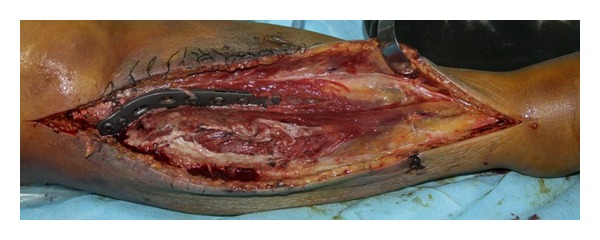
The muscle was found to be necrotic, foul smelling, and crepitant both superficially and deep.

A diagnosis of gas gangrene was made and the patient was started on broad spectrum antibiotic coverage with intravenous penicillin, clindamycin, metronidazole, and fluid resuscitation. Urgent surgery was carried out immediately. Upon incision, the musculature was found to be extensive necrotic, foul smelling, and crepitant (Figure 2). An above-the-knee amputation remained the single best life-saving treatment and was performed, followed by extensive debridement of the remaining necrotic tissue. Then she was sent to the adult intensive care unit and all the wounds were kept open postoperatively. Two days later, the patient was taken to the operating room again for wound exploration. At this time, the muscle and tissue were found to be viable without evidence of spreading infection, and the wound was closed in a standard fashion. Hemocultures and intraoperative cultures both confirmed the presence of *C. perfringens*. With aggressive surgical and medical management, this patient made a full recovery and was subsequently discharged twenty days after his initial presentation.

## 3. Reviw of Published Gas Gangrene in Orthopaedic Patients

All published, English language and full-text available, Medline-reported orthopaedic patients with gas gangrene were included in this review. Several factors, if available, were analyzed for each case: age, cause of injury, fracture location, pathogen, and clinical outcome. As seen in Table 1, we identified a total of 50 cases of gas gangrene in orthopaedic patients. Of these, 24(48%) cases were caused by *C. perfringens*. Average age is 28.75 years old (range from 5 to 76).

In our review of published orthopaedic gas gangrene literature, we found that conditions related with gas gangrene in orthopaedic patients can be grouped into three major categories: infection with clostridial myonecrosis, nonclostridial myonecrosis, unidentified; 38 patients survived, of which 25 survived with amputation, while 12 patients died. Gas gangrene followed by simple fracture occurred in 25 patients; 3 cases presented with gas gangrene after elective orthopaedic surgery and the rest cases were resulted from compound fracture. As for fracture location, most gas gangrene cases were involved with tibia and/or fibula fracture while forearm fractures ware ranked in the second place and following were femur, ankle, knee, and pelvic. Especially 5 patients developed gas gangrene with no fractures (three were selective orthopaedic surgeries, one was nail piercing, and the other one was soft tissue injury (see Table 2)).

## 4. Discussion

Gas gangrene is generally regarded as a disease associated with war or other mass casualty situations and is seldom a feature of normal peaceful time medical practice. The cause of gas gangrene could be grouped into following different types: clostridial myonecrosis, clostridial cellulitis, nonclostridial lesion simulation gas gangrene. Clostridial myonecrosis is the preferred term to denote the clinical syndrome of true gas gangrene [27]. More than 90% of these lesions occur in the extremities, thigh, shoulder, and so on. Clostridial cellulitis has been confused with clostridial myonecrosis by clinicians. Clostridial cellulitis has been noted to be a septic crepitant process involving epifascial, retroperitoneal, or other connective tissues, and its onset has been generally more gradual than clostridial myonecrosis. It is usually little pain, no edema, and little systemic toxicity. The wound is foul with brownish seropurulent exudates, and gas is found diffused through the tissues and bubbling up in the wound. The gas is much more evident than in clostridial myonecrosis, but it has never been found to be intramuscular. Also a large number of other bacterial and nonbacterial lesions which resemble clostridial myonecrosis may be seen in routine clinical practice. Many of these are diagnosed as gas gangrene and diagnostic skill knowledge is necessary for their differentiation [28]. For example, Streptococcal myonecrosis, which clinically resemble a subacute form of clostridial myonecrosis, is the second variety of anaerobic myonecrosis. Clinical considerations are listed in Table 3 when gas gangrene present.

The most common causative organism of clostridial myonecrosis is *C. perfringens* while *C. septicum* is considered as the second most frequent agent. *C. perfringens* is commonly found in the human gastrointestinal tract, including the oral cavity. Myonecrosis resulting from *C. perfringens* alone after surgical procedures is rather uncommon. Clostridium myonecrosis following orthopaedic surgery is associated with a definite set of conditions: underlying malignancies, hematological and gastrointestinal solid tumors primarily, diabetes mellitus and atherosclerotic disease, and severe peripheral vascular disease [29].

Gas gangrene is an acute and life threatening infection characterized by fever, sudden onset of prominent pain, massive local edema, severe extensive myonecrosis, and the accumulation of gas at the site of infection. The typical manifestation of this disease usually starts with excruciating pain, out of proportion to physical findings, not relieved by pain killers. As the infection progresses, myonecrosis is accompanied by necrotizing fasciitis and cutaneous and muscle necrosis. The appearance of the skin around the site of infection usually becomes tense and changes from pale to bronze initially and then to purplish red, and multiple hemorrhagic bullae develop. Paramount to successful treatment for gas gangrene involves prompt recognition of the diagnosis and initiation multiple therapy including supportive measures, antimicrobial therapy, and timely surgical intervention. Despite this, in many cases of *C. perfringens* induced gas gangrene, radical amputation still remains the treatment of best choice [30]. If not controlled, it will always result in systemic toxemia, hypotension, shock, multiorgan failure, and death [31]. Hyperbaric oxygen therapy is recommended by some experts but is controversial because its effectiveness has not yet been established.

Still we cannot identify the definitive cause of the clostridial myonecrosis in our case, as both postoperative origins and spontaneously occurrences could be possible. Our case is unique in two aspects. First, as we all know, the responsible organism *C. perfringens* is mostly associated with development of traumatic gas gangrene but also can be associated with the nontraumatic spontaneous gas gangrene in patients with immunocompromised condition including malignancies and diabetes mellitus [2, 32]. Impaired evacuation and motility of the stomach (and the small intestine) has been described in diabetics with long lasting unsatisfactory diabetes compensation, microangiopathic complications, and diabetic autonomous neuropathy [33]. Postoperative infection of elective surgical wounds with Clostridium species has been linked to gastrointestinal tract lesions. As clostridia can multiply readily in low-oxygen conditions, infections are usually seen in the setting of decreased intestine lining blood supply which could account for a route of entry for hematogenous spread. Second, gas gangrene rarely occurs in the patients undergoing elective surgery. One of the basic principles of orthopedic surgery is that gas gangrene does not develop in closed fractures. Almost all cases of gas gangrene after orthopaedic surgery developed in open wounds which was not adequately debrided, in association with peripheral vascular disease and immunocompromised status. Even patients with closed fracture clostridium gas gangrene also had been found [11]. In our case, a possible mechanism is soil contamination of the skin near or at the infection site, as well as the severe injured soft tissue around the fracture together that contribute to the production histohypoxia environment. All these factors such as immunocompromised status, unviability of tissues, and local decrease of blood supply together nourished the gas gangrene. But the exact origin of the germ remains unknown.

## 5. Conclusion

Based on the case presented in the paper and our review of the literature on gas gangrene in orthopaedics patients, several following points should be emphasized.

1. Our emergency clinicians should be aware of this severe and potentially fatal infectious disease and should not delay treatment or prompt orthopedic surgery consultation. Gas gangrene, while rare in now peace days, can be a devastating complication of almost any small wound or surgical procedure even one as common as closed reduction of fractures. It is our experience that we should give sufficient extension of the wound to provide adequate visualization of surgical field so as to be certain that all the necrotic or foreign material has been removed.
2. Strict aseptic techniques should be observed for even the most minor procedure. Clostridial spores are ubiquitous and can reside in hospital environments, possibly on surgeons' hands, patients' skin, topical application, and so on.
3. The best way to prevent gas gangrene is meticulous wound debridement and delayed closure for all potentially contaminated wounds regardless of closed or open fractures.
4. Once gas gangrene is diagnosed, careful and adequate debridement should be instituted immediately to avoid further deterioration excision of necrotic tissue still the cornerstone of treatment, which should be involved with antibiotics and all other supportive treatments.
5. Systematic resuscitative efforts should be instituted immediately in whom the diagnosis of incipient gas gangrene is even considered. This cannot be overemphasized.
6. Recognized that gas gangrene may occur spontaneously and often in a immunocompromised patient, postoperative wounds may also develop gas gangrene due to the local soft tissue damage and decreasing blood supply.

## Figures and Tables

**Table 1:** Gas gangrene infections in traumatic orthopaedic patients.

Source	Cause	Age	Fracture	Soft tissue	Pathogen	Outcome
Fee, 1977 [5]	Fall from a tree	8	Closed, forearm	A small laceration	Gram-positive Spore-forming rods	Lived with disarticulation
	Fall from a tree	10	Open, right forearm	Two small lacerations	*Clostridium perfringens *	Lived with amputation
	Fall from a tree	11	Open, right forearm	A puncture wound	*Clostridium perfringens *	Lived with amputation
	Fall from a roof	12	Open, right forearm	Two openings	*Clostridium perfringens *	Lived with preserved forearm
	Fall on the street	52	Open, left radius	A puncture wound	Gram-positive rods	Lived with amputation

Buchanan and Gordon, 1980 [6]	Fall from 5 stories	19	Compound fracture of right tibia and fibula	Contaminated with dirt, gravel	*Clostridium perfringens *	Lived with amputation

Fulford, 1969 [7]	Traffic accident	19	Open, right femur	Contaminated, No detail	Unknown	Lived with preserved limb

Lucas et al., 1976 [8]	Kicked on right shin in a tackle	24	Tibia and fibula	7 cm wound	*Clostridium septicurn *	Lived with preserved limb

Woolley et al., 2004 [9]	Fall from high place	39	Open, left tibia, and fibula	Gustillo Grade III	*Clostridium septicurn* (no gas gangrene)	Lived with preserved limb

Werry and Meek, 1986 [10]	Unknown	32	Distal radius	Abrasion of the volar wrist skin	*Clostridium perfringens *	Lived with amputation

Goon et al., 2005 [11]	Local accident	76	No fracture	No traumatic history	*Clostridium septicurn*	Died

Taylor et al., 2011 [12]	High-speed motor vehicle collision	21	Closed, right femur with traction pin	Multiple organ injuries	*Clostridium perfringens *	Died

Mulier et al., 1993 [13]	Fall from a height of 8 feet	45	Closed, femoral fracture	Unknown	*Clostridium septicurn*	Survived with disarticulation

Lorea et al., 2004 [14]	Muscle transfer for opponensplasty	49	No fracture	Normal muscle surgery	*Clostridium perfringens*, Sordellii	Survived with preserved forearm

Sevitt, 1953 [15]	Playing football	26	Closed, left ankle	Unknown	*Clostridium perfringens *	Survived with amputation

HILL, 1959 [16]	Fall from a gate	6	Left forearm fracture	A small wound on the forearm	Unknown	Survived with amputation

Aufranc et al., 1969 [17]	Struck by a rotating truck wheel	29	Open, right tibia	Wringer-type injury of leg.	Presence of gram-positive rods	Survived with preserved limb

	Automobile accident	13	Compound, left tibia, and fibula	Unknown	Bacilli welchii	Died
	Fall from window to ground	24	Compound, left tibia	Unknown	Bacilli welchii	Survived with amputation
	Automobile accident	37	Compound, left tibia, and fibula	Unknown	Bacilli welchii	Died
	Hooked by a cow	6	Compound, right forearm	Unknown	Bacilli welchii	Survived with amputation
Boland, 1929 [18]	Street accident	52	Compound, left tibia, and fibula	Unknown	Positive culture, detail Unknown	Survived with amputation
	Truck Accident	21	Compound, left tibia, and fibula	Unknown	Positive culture, detail Unknown	Survived with preserved limb
	Motorcycle accident	18	Compound, right tibia, and fibula	Unknown	Bacilli welchii	Survived with amputation
	Struck by a truck	5	Compound, upper extremity, left femur, left tibia, and fibula	Unknown	Bacilli welchii	Died
	Street-car accident	13	Compound, both legs	Unknown	Bacilli welchii	Died
	Knee joint fracture from gun-shot	30	Knee joint fracture	Unknown	Positive culture, detail Unknown	Survived with amputation
	Auto accident	36	Compound, right tibia, and fibula	Unknown	Positive culture, detail Unknown	Survived with amputation
	Falling from freight train	16	Compound, both legs	Unknown	Positive culture, detail Unknown	Survived with amputation
	Motorcycle accident	40	Compound, left tibia, and fibula	Unknown	Positive culture, detail Unknown	Survived with preserved limb
	Automobile accident	25	Compound, both legs	Unknown	Positive culture, detail Unknown	Died
	Gun-shot	20	Compound, right ulnar, and radius	Unknown	Negative culture	Died

Brume and Ijagha, 1985 [19]	Unknown	9	Closed Colles' Fracture	Unknown	Unknown	Survived with amputation
	Unknown	27	Closed, medical malleolus	Unknown	Unknown	Survived with amputation
	Unknown	30	Closed,tibia and fibula	Unknown	Unknown	Died
	Unknown	14	Closed Colles' Fracture	Unknown	Unknown	Survived with amputation

Moehring, 1988 [20]	Automobile accident	13	Right ankle region	Marked soft tissue swelling	*Clostridium perfringens *	Lived with preserved limb

Oncel and Arsoy, 2010 [21]	Nail pierced the skin of hand	16	No fracture	A small wound on the hand	Gram-positive rods	Survived with amputation

Hoffman et al., 1971 [22]	Working accident	25	Tibia fracture	Muscle and skin lacerated	Clostridium welchii	Survived

DeHaven and Evarts, 1971 [23]	Fall from horseback	10	Open, both bones of forearms	Mild damage of soft tissue	*Clostridium perfringens *	Survived with amputation
	Automobile accident	44	Open, Bilateral tibia, and fibulae	Damaged and contaminated severely	Pseudomonas, Klebsiella et al.	Survived with amputation
	Automobile accident	21	Open, pelvic	Multiple	*Clostridium perfringens *	Survived
	Fall from running	10	Open, both bones of forearms	Mild damage of soft tissue	*Clostridium perfringens *	Survived with amputation
	Automobile accident	19	Open, tibia, and fibula	Severe damage of soft tissue	Bacillus subtilis, Proteus	Survived with amputation

Johnson et al., 1994 [24]	Arthroscopic knee surgery	36	No fracture	No	*Clostridium Septicum*	Survived with amputation
	Hip Arthroplasty	57	No fracture	No	*Clostridium Septicum*	Survived

Dykes, 1977 [25]	Hip nailing	71	Transcervical fracture of femur	No	Clostridium welchii	Died
	Hip nailing and plate fixation	68	Subtrochanteric fracture of femur	No	Unknown	Died
	Hip nailing	79	Transcervical fracture of femur	No	Unknown	Died
Miller et al., 1993 [26]	Iliac crest bone graft transplantation	55	Nonunion of closed fracture of clavicle	No	*Clostridium perfringens *	Survived

(1) Compound fracture indicates open fracture, while simple fracture means closed fracture.

(2) Bacillus welchii is another expression of *Clostridium perfringens*.

(3) *Clostridium perfringens* (formerly known as *C. welchii*).

**Table:** (a) Clinical outcome after infection with gas gangrene

Outcome	Survived with amputation	Survived with no amputation	Died
Number	25	13	12

**Table:** (b) Pathogen of gas gangrene accompanied with traumatic orthopaedic patients

Pathogen	*Clostridium myonecrosis *	Non-clostridial myonecrosis	Unidentified
Number	28	3	19

*Clostridium myonecrosis* including Clostridium perfringen and septium while nonclostridial myonecrosis including culture negative; unidentified indicates no detail about the infection pathogen were reported.

**Table:** (c) Fractures locations together with gas gangrene

Location	Tibia and/or fibula	Forearm	Femur	Ankle	Clavicle	Knee	Pelvic	None
Number	21	14	7	2	1	1	1	S

One case involved multiple locations of fractures: forearm, tibia and fibula, and femur; forearm including both or single bones of the forearm.

**Table:** (d) Fractures or surgeries types associated with gas gangrene

Fracture severity	Simple fracture	Compound fracture	Elective orthopaedic surgery
Number	25	22	3

Compound (open) fracture: the bone breaks and pieces of the bone go through the internal soft tissue of the body and break through the skin from the inside.

**Table 3:** Clinical consideration when gas gangrene is present.

(I) Clostridial myonecrosis (true gas gangrene)	
(A) Localized: crepitant or noncrepitant	
(B) Diffuse: crepitant or noncrepitant together with toxemia	
(II) Clostridial cellulitis: anaerobic or crepitant	
(III) Nonclostridial	
(A) Bacterial: aerobic aerogenic infections; Staphylococcal fasciitis; anaerobic streptococcal infections	
(B) Nonbacterial: mechanical trauma; infiltration from air-hose injury	
